# Both *Penicillium expansum* and *Trichothecim roseum* Infections Promote the Ripening of Apples and Release Specific Volatile Compounds

**DOI:** 10.3389/fpls.2019.00338

**Published:** 2019-03-20

**Authors:** Di Gong, Yang Bi, Yongcai Li, Yuanyuan Zong, Ye Han, Dov Prusky

**Affiliations:** ^1^College of Food Science and Engineering, Gansu Agricultural University, Lanzhou, China; ^2^Department of Postharvest Science of Fresh Produce, Agricultural Research Organization, Volcani Center, Rishon LeZion, Israel

**Keywords:** apple, fungi, physiology, quality, volatile compounds

## Abstract

Blue mold and core rot caused by *Penicillium expansum* and *Trichothecium roseum* are major diseases of apple fruit in China; however, their differential aggressiveness in apples and effect on fruit postharvest physiology are unclear. The effects of colonization of apples cv. Red Delicious by both pathogens were compared to physiological parameters of ripening and release of volatile compounds (VOCs). *P. expansum* colonization showed increased aggressiveness compared to *T. roesum* colonization of apple fruits. *P. expansum* enhanced colonization occurred with differential higher ethylene production and respiratory rate evolution, lower membrane integrity and fruit firmness in correspondence with the colonization pattern of inoculated apples. Moreover, *P. expansum* caused lower contents of total soluble solid and titratable acid, and higher malondialdehyde compared with *T. roesum* colonization. While both pathogen infections enhanced VOCs release, compared with *T. roseum* inoculated apples, *P. expansum* inoculated apple showed a higher total VOCs production including alcohols, aldehydes and esters, being the C6 alcohols, aldehydes and esters amount. PLS-DA analysis indicated that hexanoic acid was the most important factor to distinguish the inoculated fruits from the controls. Interestingly, propyl acetate and hexyl benzoate, and undecylenic acid and hexadecane were only identified in the *P. expansum* and *T. roseum* inoculated fruits, respectively. Taken together, our findings indicate that both fungi inoculations promote apple fruit ripening and release specific VOCs; moreover, apple fruits are more susceptible to *P. expansum* colonization than *T. roesum*.

## Introduction

Apple is one of the most important temperate fruit produced in China, which is the largest apple producing and consuming country in the world (Sharma et al., 2014). However, apple fruit is susceptible to postharvest fungi, among them, *Penicillium expansum* and *Trichothecim roseum*, the causal agents of blue mold and core rot, are the major fungi in China (Quaglia et al., 2011; Xue et al., 2017). *P. expansum* infects fruit primarily through wounds caused by stem punctures and bruises occurring at harvest or during postharvest handling (Vilanova et al., 2017). While *T. roseum* enters the fruit through natural openings, the mycelia grows in the locules of fruit, then penetrates into the mesoderm leading to rot of flesh (Niu et al., 2015). Both fungi produce patulin and trichothecenes in infected fruit, which pose health risk for consumers (Al-Rawashdeh and Karajeh, 2014; Xue et al., 2017).

Increasing of ethylene release and respiration rate is a typical harvested fruit response to fungi infection (Rojas et al., 2014), which has been observed in various fruit–fungi interactions, including apple-*Penicillium digitatum* (Vilanova et al., 2017), pear-*Botrytis cinerea* (Hundalani and Pammi, 2007), citrus-*P. digitatum* (González-Candelas et al., 2010; Marcos et al., 2005), grapes-*B. cinerea* (Zhu et al., 2012), and banana*-Colletotrichum musae* (Daundasekera et al., 2008). Fungal infections also accelerate fruit quality decline as reported for *B. cinerea* infection that reduced firmness of pear fruit (Hundalani and Pammi, 2007). Similarly the level of total soluble solid (TSS) and titratable acid (TA) declined in *P. expansum* colonized apples (Da et al., 2016) In addition, these changes were accompanied by decrease membrane permeability and cause oxidative stress of fruit (Yi et al., 2009).

In apple, volatile compounds (VOCs) that contribute to aroma are important components of fruit consumer appeal (Lumpkin et al., 2015). Pathogen infections not only are significant responsible for quality losses, but also affect VOCs release of fruit (Vikram et al., 2004a,b; López et al., 2015; Encinas-Basurto et al., 2017). *P. expansum* inoculated apples were showed to enhance VOCs release from “McIntosh” and “Cortland” apple fruits. Contents of ester increased, while the contents of alcohol and aldehyde were declined in infected fruit (Vikram et al., 2004a,b). In addition, some specific VOCs such as (*Z*)-3-hexenyl 2-methylbutanoate was identified only in specific colonized apple cultivars infected by *P. expansum* and *Rhizopus stolonifer*, and 2-butanone and α-pinene were only detected in *R. stolonifer*-infected “Blanquilla” pears (López et al., 2015).

However, little information is known about the effect of *T. roseum* on physiology, quality and VOCs of apple fruit. In addition, little is known regarding different effects between *P. expansum* and *T. roseum* infections. The objectives of this study are to evaluate the effect of two fungi infections on ripening and quality of apple fruit, especially, to understand VOCs releasing from the fruit response to the two fungi infections.

## Materials and Methods

### Plant Material

Apple fruit (*Malus domestica* L. cv Red Delicious) were obtained from a commercial orchard in Jingtai County, Gansu province of China, in October, 2017. Fruit were harvested at physiological maturity (firmness was 17.68 ± 0.89 N and TSS content was 14.94 ± 0.72%) and screened for uniform size and lack of visible defects. 300 fruits were packed individually in net bags of foamplastic, placed in the apple shipping boxes (50 apples per box), then transported to the laboratory on the same day. Upon arrival fruit was surface-sterilized in 0.2% sodium hypochlorite solution for 2 min, washed with sterile distilled water and allowed to dry at room temperature.

### Pathogens and Spore Suspension

*Penicillium expansum* was from the Institute of Botany, Chinese Academy of Sciences; and *T. roseum* was from the Department of Postharvest Biology and Technology group of Gansu Agricultural University. Conidia of the two fungi were from 7-day-old PDA cultures incubated at 25°C. The cultures were flooded with sterile water containing 0.05% Tween-80 and rubbed gently with a glass rod, filtered through two layers of sterile cheesecloth. Concentration of each fungus was determined using a haemocytometer and prepared to obtain 1 × 10^6^ conidia mL^-1^ of them.

### Fruit Inoculation

Apples were wounded by making 2 injuries (1 mm width, 2 mm depth) with a nail on two opposite sides of each fruit at the central equatorial region of the fruit, and 20 μL spore suspension of each fungus was placed into each wound. Non-wounded apples were used as healthy the control tissue (N-control), while wounded apples but inoculated with 20 μL sterile water containing 0.05% (w/v) Tween-80 were used as mock-inoculation (W-control). After 2 h of air-dry, the fruits were put in carton boxes without fruit touched, and each box was covered with plastic film to maintain a relative humidity (80–90%) and stored at 20 ± 1°C for assaying.

### Sampling

Flesh tissue samples from the healthy part of the fruit taken along the equator of fruit at 2–3 mm below the skin and 1 cm away from the edge of the decay tissue for TA and malondialdehyde (MDA) analysis. The tissue samples were frozen in liquid nitrogen and stored at -80°C until analyzed. Moreover, peel tissue samples from the healthy part of the fruit taken along the equator of fruit and 1 cm away from the edge of the decay tissue were taken and stored at -20°C for VOCs analysis. Each treatment contained sixty apple fruits. Twelve of them, as experimental units, were evaluated at 0, 2, 4, 6, and 8 days after inoculation (DAI). Each experiment was repeated three times over time.

### Determination of Lesion Diameter

The lesion diameters (cm) of the same fruit were measured at 0, 2, 4, 6, and 8 DAI using method of Da et al. (2016) with modified. Two diameter values of each lesion in two mutually perpendicular directions were recorded by a standard ruler on every sampling day. The average of the two values was defined as the diameter of the lesion.

### Determination of Respiration Rate and Ethylene Release

Ethylene production of the same intact fruit was measured at 0, 2, 4, 6, and 8 DAI by gas chromatography (Agilent Technologies 7820A, Beijing Keep-Science Analysis Sci & Tech Co., LTD) equipped with thermal conductivity and flame ionization detectors. Fruits were placed individually in airtight jars capped with a rubber stopper for 6 h, at 25°C. 0.2 mL of headspace gas was sampled and used to detect ethylene production (Zhang et al., 2009). Production was expressed as C_2_H_4_ concentration (μg kg^-1^ h^-1^).

Respiration rate of the same intact fruit was measured at 0, 2, 4, 6, and 8 DAI by Fruits and Vegetables Respiration Apparatus (JFQ-315OH, Jun-Fang-Li-Hua Technology Institute, Beijing, China). Fruits were placed individually in airtight jars and the valve was opened to let air in. Production was expressed as CO_2_ concentration (mg kg^-1^ h^-1^).

### Determination of Membrane Integrity and Malondialdehyde (MDA) Content

Membrane integrity was expressed using the relative leakage rate and was measured using the method of Zhang et al. (2017) and modified. Healthy tissue disks of nine fruit were taken at 1 cm away from decay tissue were derived from twelve fruits with a cork borer (3 mm in diameter), washed twice, and incubated in 40 ml of distilled water at 25°C for 3 h. The initial electrolyte value of the bathing solution was determined using a conductivity meter (DDSJ-308A, INESA Analytical Instrument Co., Ltd, Shanghai, China). After the 3 h of incubation, the solution with disks was boiled for 30 min, quickly cooled, and replenished with distilled water to 40 ml, and the total electrolyte leakage of the solution was again measured. Relative leakage rate was expressed as the percentage (%) of the electrolyte value obtained following the 3 h incubation relative to the total electrolyte content after boiling.

Malondialdehyde content was measured using the method of Zhang et al. (2015) and modified. 3 g frozen apple flesh tissues were ground by liquid nitrogen, then the powder was homogenized in 5 ml of 100 g L^-1^ trichloroacetic acid (TCA) and centrifuged at 12000 *g* for 20 min at 4°C. 2 ml supernatant (2 mL TCA as control) was mixed with 2 ml of 0.67% (w/v) thiobarbituric acid (TBA) and incubated for 20 min in a boiling water bath, cooled quickly in an ice bath and then centrifuged at 12000 *g* for 20 min at 4°C. The supernatant was measured at recorded at 600, 532, and 450 nm using ultraviolet spectrophotometry. The MDA contents of reaction solution and samples were calculated according to the following formulas. The MDA content was expressed as nmol g^-1^ FW^-1^.

$$\mathrm{MDA}\;\mathrm{content}\;\mathrm{of}\;\mathrm{reaction}\;\mathrm{solution}(C_{\mathrm{MDA}})\left(\frac{\mathrm{\mu mol}}{L}\right)=6.45\times ({\mathrm{OD}}_{532}-{\mathrm{OD}}_{600})-0.56\times {\mathrm{OD}}_{450}$$

$$\mathrm{MDA}\;\mathrm{content}\;\mathrm{of}\;\mathrm{samples}\;(\mathrm{nmol}/g\cdot \mathrm{FW})=C_{\mathrm{MDA}}\left(\frac{\mathrm{\mu mol}}{L}\right)\times \frac{\mathrm{volum}\;\mathrm{of}\;\mathrm{extraction}\;\mathrm{solution}\;(\mathrm{ml})}{\mathrm{fresh}\;\mathrm{weight}\;\mathrm{of}\;\mathrm{fruit}\;\mathrm{tissue}\;(g)}$$

### Determination of Firmness, TSS, and TA

Firmness of healthy tissue that 1 cm away from decay tissue was measured using a hand-held fruit firmness tester (GY-1, Hangzhou, China) at the equator of the fruit (Zhang et al., 2011). Four readings were taken for each fruit.

For TSS and TA analysis, 10 g frozen apple flesh tissues were ground, then constant volume to 100 ml with distilled water for TA measurements. 10 mL of fruit juice were taken and titrated with 0.2 M NaOH until reaching a pH of 8.2. TSS values of fresh healthy tissue that 1 cm away from decay tissue measured with a digital refractometer (TD-45, Hangzhou, China) (Lee et al., 2017).

### Volatile Compound Analysis

The concentrations of VOCs were determined according to Xi et al. (2016) with some modifications. Peels of twelve apple fruits were ground into a fine powder in liquid nitrogen, then 5 g powder was quickly transferred into a 10 mL headspace bottle containing 1.0 g of NaCl and 10 μL 3-Nonanone (0.04 μL mL^-1^) as an internal standard, then equilibrated in a laboratory stirrer/hot plate (model PC-420, Corning Inc., Life Science, Acton, MA, United States) at 40°C for 30 min. Volatiles were extracted using 50/30 μmpolydimethylsiloxane/divinylbenzene/carboxen fiber (PDMS/DVB/CAR) (Supelco, Inc., Bellefonte, PA, United States).

For quantitation of VOCs, the solid-phase micro-extraction (SPME) device was inserted into the injection port of Thermo Scientific 265079 equipped with a TG-WAX column (60 m × 0.32 mm with a 1 mm film thickness) (Thermo Fisher Scientific, United States) was used to analyzed VOCs. The injection port temperature was 250°C. Helium was used as the carrier gas at a rate of 1.0 mL min^-1^. The GC oven temperature was held at 40°C for 3 min, increased by 5°C min^-1^ to 150°C, and increased by 10°C min^-1^ to 250°C and then held for 5 min. Mass spectra were obtained by electron ionization at 70 eV and a scan range of 20–500 mass units.

Compounds were identified by comparing the spectra with the NIST-98/Wiley library and by matching retention index of authentic reference standards. Quantitative determination of volatiles was calculated based on internal standard.

### Statistical Analysis

Statistical analysis was performed with SPSS version 19.0 (SPSS Inc., Chicago, IL, United States). Results were presented as mean value ± standard errors. To determine the effect of treatments, the data were compared in a Duncan’s test. Differences at *P* < 0.05 were considered as significant. Heatmap was employed to characterize the relative levels of GC-MS data throughout all sample groups. Partial least squares discriminant analysis (PLS-DA) was used for a supervised analysis. The GC-MS data variables were normalized using Metaboanalyst version 4.0^1^ by mean-centered and divided by the standard deviation of each variable before developing heatmap and PLS-DA model. All of the different metabolites were identified and characterized using the loading plots and VIP value (cut-off > 1) from the PLS-DA analysis.

## Results

### Changes in Lesion Diameter, Ethylene Production and Respiratory Rate of Fruit After Two Pathogens Inoculation

**FIGURE 1:**
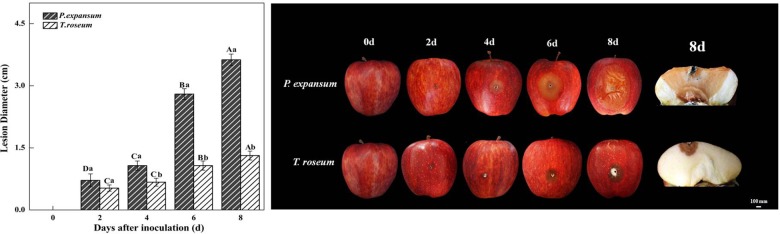
Effects of *Penicillium expansum* and *Trichothecim roseum* inoculation on lesion diameter of apple fruit during storage. All data are expressed as means ± standard deviation of triplicate samples. Different letters indicate significant differences (*P* < 0.05). The picture on the right shows the lesion diameter of fruit inoculated with the two pathogens during storage.

Fruit inoculated with *P. expansum* and *T. roseum* exhibited different rates of symptoms during colonization (Figure 1). Measurement of lesion diameters on the inoculated fruits showed that *P. expansum* caused larger lesion diameter during storage, which was 177% larger than *T. roseum* at 8 DAI (*P* < 0.05) (Figure 1). *P. expansum* decay lesions showed a small and light tan, and no apparent sporulation was observed in the rotten tissue at 2 DAI. After 4 DAI, the lesions became larger and visible blue-green spores were found around wounded hole. The lesion diameter of the *P. expansum*-inoculated fruit was 0.71 cm at 2 DAI and reached to 3.63 cm at 8 DAI. The symptoms on *T. roseum*-colonizing apple were brown rot at 2 DAI. After 4 DAI, the lesions became larger and visible pinkish spores were found on rotted tissue. The lesion diameter of the *T. roseum*-inoculated fruit was 0.53 cm at 2 DAI and reached to 1.31 cm at 8 DAI.

**FIGURE 2:**
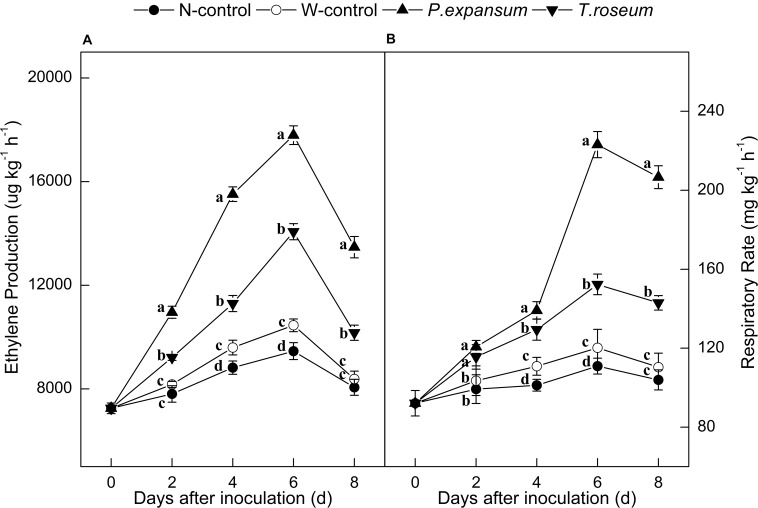
Effects of *P. expansum* and *T. roseum* inoculation on ethylene production **(A)** and respiratory rate **(B)** of apple fruit during storage. All data are expressed as means ± standard deviation of triplicate samples. Different letters indicate significant differences (*P* < 0.05).

Ethylene production in the inoculated fruits increased sharply at 6 DAI and decreased thereafter (Figure 2A). Both *P. expansum* and *T. roseum* inoculations significantly induced higher peaks of ethylene production, which were 88 and 49% higher than that in the N-control (*P* < 0.05). Compared with the *T. roseum-*inoculated fruit, higher ethylene production was showed in the *P. expansum*-inoculated fruit during storage and the peak value was 26% higher than the *T. roseum* inoculated ones (*P* < 0.05).

Single peaks of respiratory rate were observed in the treated fruit at 6 DAI (Figure 2B). The respiratory rate was also significantly increased by the both inoculations, compared with N-control, the peak values of respiratory rate were 102 and 37% higher in the *P. expansum* and *T. roseum* inoculated fruits (*P* < 0.05). Compared with the *T. roseum*-inoculated fruit, higher respiratory rate was observed in the *P. expansum*-inoculated fruit and the peak was 46% higher in the *P. expansum*-inoculated ones (*P* < 0.05). Nevertheless, ethylene production and respiratory rate of W-control significantly increased during its climacteric stage, but the trend was lower than the colonized tissue.

### Changes in Membrane Integrity and MDA Content of Fruit After Two Pathogens Inoculation

**FIGURE 3:**
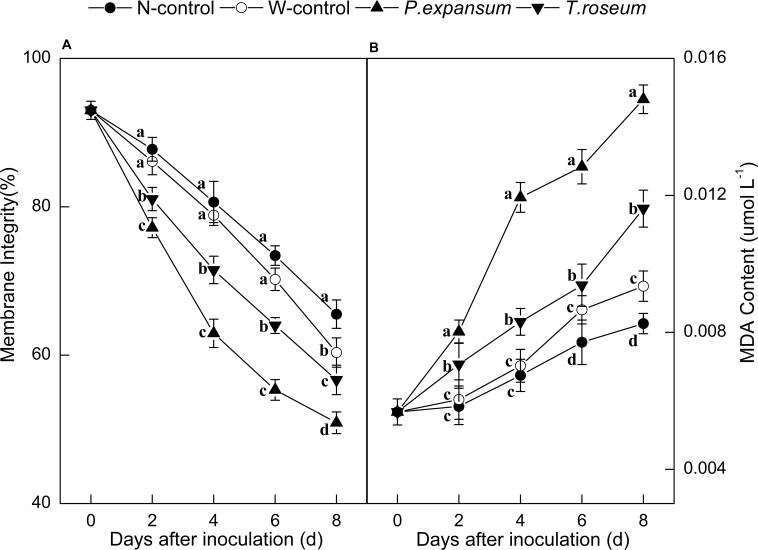
Effects of *P. expansum* and *T. roseum* inoculation on membrane integrity **(A)** and MDA content **(B)** of apple fruit during storage. All data are expressed as means ± standard deviation of triplicate samples. Different letters indicate significant differences (*P* < 0.05).

Compared with the controls, inoculations with the two pathogens significantly decreased membrane integrity of fruit (Figure 3A). The membrane integrity of the *P. expansum* and *T. roseum*-inoculated fruits was 22 and 14% lower than that of the N-control (*P* < 0.05). Moreover, the *P. expansum*-inoculated fruit showed lower membrane integrity than the *T. roseum*-inoculated one, which was 10% lower at 8 DAI (*P* < 0.05). Membrane integrity of W-control was 8% lower than that of the N-control at 8 DAI (*P* < 0.05). In addition, MDA contents of fruits were dramatically promoted by the two pathogens inoculation (Figure 3B). The MDA contents of the *P. expansum* and *T. roseum* inoculated fruits were 78 and 45% higher than that in the N-control at 8 DAI (*P* < 0.05). Compared with the *T. roseum* inoculated fruits, higher MDA content was showed in the *P. expansum*-inoculated fruit, which was 28% higher at 8 DAI (*P* < 0.05). Moreover, MDA content of W-control showed 13.20% higher than that in N-control at 8 DAI (*P* < 0.05).

### Changes in Firmness, TSS, and TA Contents of Fruit After Two Pathogens Inoculation

**FIGURE 4:**
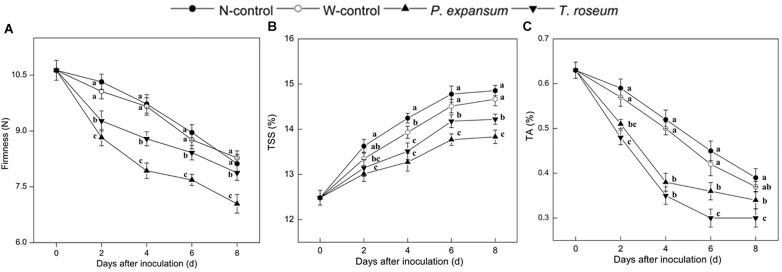
Effects of *P. expansum* and *T. roseum* inoculation on firmness **(A)**, TSS **(B)**, and TA content **(C)** of apple fruit during storage. All data are expressed as means ± standard deviation of triplicate samples. Different letters indicate significant differences (*P* < 0.05).

Compared with the controls, firmness was significantly decreased by the two pathogens inoculations during storage (Figure 4A). The firmness of the *P. expansum* and *T. roseum*-inoculated fruits was 19 and 10% lower than that in the N-control at 4 DAI (*P* < 0.05). Compared with the *T. roseum-*inoculated fruit, firmness was lower in the *P. expansum*-inoculated ones, which was 11% lower at 8 DAI (*P* < 0.05). In addition, the firmness of control fruits both in non-wound and wounded had no difference during storage.

Contents of TSS and TA significantly decreased after two pathogens inoculation (Figure 4B,C). TSS content of the treated fruits showed single peak change at 4 DAI. *P. expansum* and *T. roseum*-inoculated fruits maintained lower than the controls during storage, which were 11 and 6% lower than that in the N-control at 6 DAI (*P* < 0.05). Compared with the *T. roseum*-inoculated fruit, TSS content in the *P. expansum*-inoculated ones was 5% lower at 6 DAI (*P* < 0.05). As regards to TA contents, *P. expansum* and *T. roseum* inoculation promoted the decrease of TA contents, which were 33 and 20% lower than that in the N-control at 6 DAI (*P* < 0.05). Moreover, the *P. expansum*-inoculated fruit had lower TA content than that in the *T. roseum*-inoculated ones during storage, which was 17% lower at 6 DAI (*P* < 0.05). Furthermore, wounded fruit (W-control) also showed decreased in the contents of TSS and TA of W-control fruits showed decreased as compared with non-wound ones, but it had no significant effect on them.

### Changes in VOCs of Fruit After Two Pathogens Inoculation

#### Volatile Compounds Emission Detected

**FIGURE 5:**
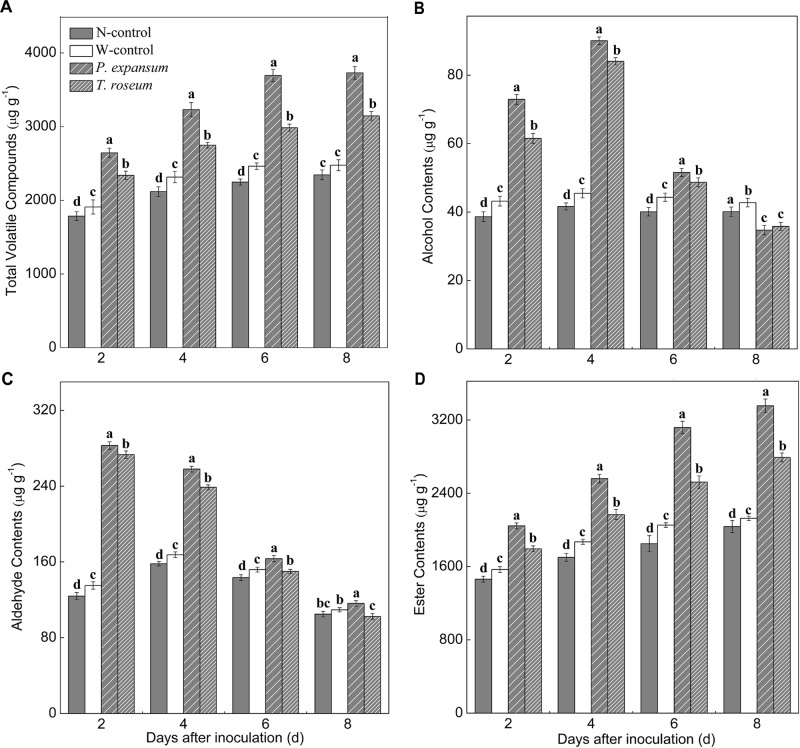
Effects of *P. expansum* and *T. roseum* inoculation on contents of total VOCs **(A)**, alcohol **(B)**, aldehyde **(C)**, and ester **(D)** in apple fruit during storage. All data are expressed as means ± standard deviation of triplicate samples. Different letters indicate significant differences (*P* < 0.05).

The VOCs emitted at 0, 2, 4, 6, and 8 d were quantified after two pathogens inoculation and grouped into chemical classes, including alcohol, aldehyde, and ester (Figure 5). Content of total VOCs in the treated fruit showed increased trend during storage, and the inoculated fruits showed higher content of total VOCs than controls due to increased ester production (Figure 5A). Total VOCs contents of *P. expansum* and *T. roseum*-inoculated fruits were 59 and 34% higher than that in N-control. Moreover, *P. expansum*-inoculated fruit maintained higher content of total VOCs than the *T. roseum*-inoculated ones, which was 18% higher at 8 DAI. Esters were the predominant volatile class in the treated fruit, which showed increased trends after inoculation (Figure 5D). Esters of *P. expansum* and *T. roseum*-inoculated fruits were 65 and 37% higher than that in the N-control at 8 DAI. Compared with the *T. roseum*-inoculated ones, esters of *P. expansum*-inoculated fruit was 20% higher during storage. Contents of alcohol and aldehyde were increased in the treated fruit in the first 4 days and then decreased (Figure 5B,C). Alcohols and aldehydes in the *P. expansum*-inoculated fruits showed 106 and 63% higher than in the N-control ones. Similarly, both of them in the *T. roseum*-inoculated fruits showed 102 and 51% higher than in the N-control ones. Additionally, the VOCs released in control fruits (non-wound or wounded) was no significant change during storage.

Volatile compounds were identified in the treated apples including 2 alcohols, 2 aldehydes, 32 esters, 3 terpinoides and 8 others (3 acids, 4 alkanes and 1 aromatics) (Table. 1). The number of VOCs in the N-control was 43, and the W-control and both of inoculated ones yielded 42 compounds. Compared with N-control and W-control, propyl acetate and hexyl benzoate were only identified in the *P. expansum*-inoculated fruits, and undecylenic acid and hexadecane were only identified in the *T. roseum*-inoculated ones. Nevertheless, both of inoculations had no significant effect on number of VOCs, but it affected the concentration range of VOCs. The *P. expansum*-inoculated fruit showed the largest rang of concentrations (0–1012.97 μg g^-1^). While *T. roseum*-inoculated, N-control and W-control ones showed range of concentrations from 0.46 to 911.54 μg g^-1^, 0.78 to 757.08 μg g^-1^, and 0.73 to 683.19 μg g^-1^, respectively.

**FIGURE 6:**
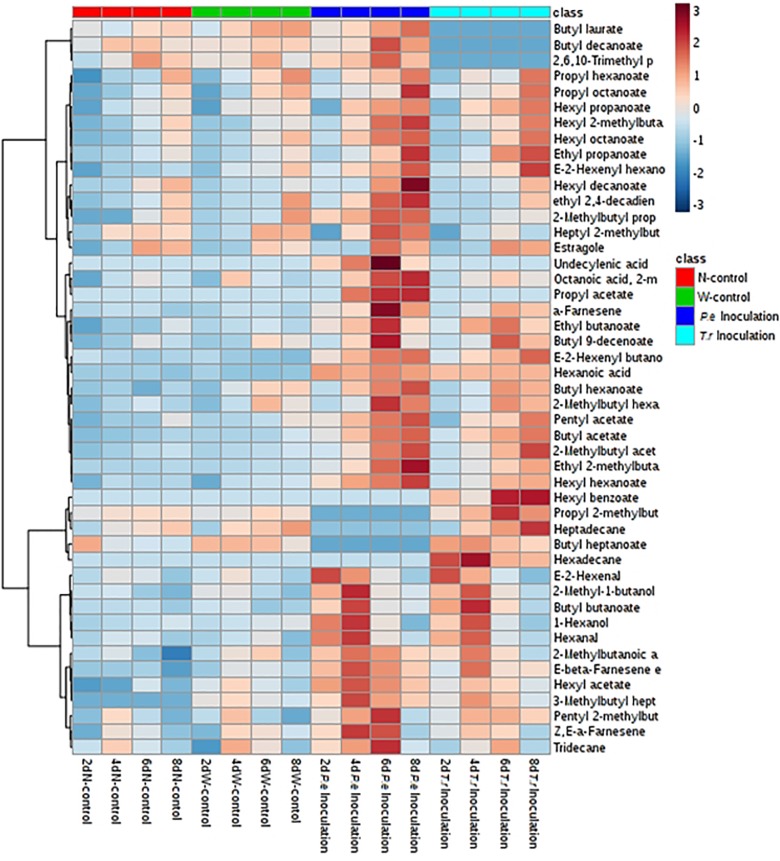
Heatmap cluster based on the normalized quantities of the identified volatiles in apple fruits. Each line in the heatmap represents a volatile compound. The lowest content is in the darkest blue and the highest content is in the darkest red.

It was obviously that most of VOCs changed after two pathogens inoculation during storage, especially C6 VOCs (Figure 6). Compared with N-control and W-control, C6 aldehydes and alcohols were increased after stimulated by two pathogen at early inoculation stage (2 and 4 DAI), including 1-hexanol, hexanal and (*E*)-2-hexenal. What’s more, C6 aldehydes and alcohols contents in the *P. expansum*-inoculated ones were higher than that in the *T. roseum*-inoculated ones. Nevertheless, esters, derived from C6 alcohols, were obviously stimulated by two of them inoculation during storage, especially in the last 4 days, hexyl acetate, hexyl propanoate, hexyl hexanoate, hexyl octanoate, propyl hexanoate, butyl hexanoate, hexyl 2-methylbutanoate and 2-methylbutyl hexanoate were significantly increased. Moreover, contents of these esters in the *P. expansum*-inoculated ones remained higher than that in the *T. roseum*-inoculated ones during storage.

#### PLS-DA Analyzed Volatile Metabolites

**FIGURE 7:**
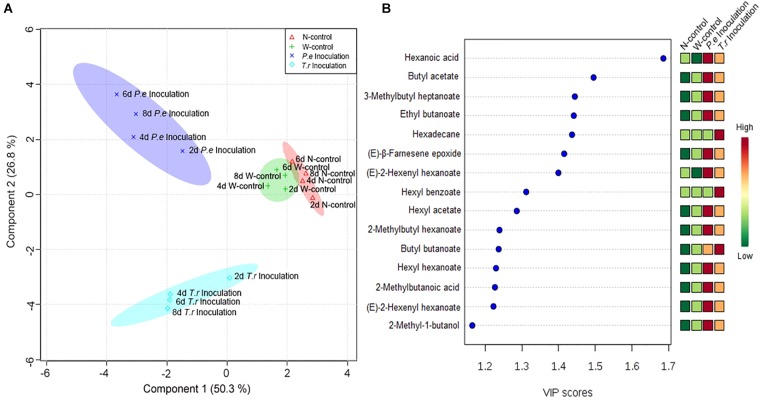
PLS-DA analysis **(A)** and associated VIP score **(B)** were observed in the PLS-DA models of apple fruit during storage. **(A)** Score-plot of PLS-DA analysis according to identified volatiles in apple fruits. The markers with different shapes represent volatile samples of apple fruits from different treatments. Different color spaces represent a group and show every 2 days variation in four treatments apple fruit. **(B)** VIP score cut-off at 1.

The quantitative data of the identified VOCs subjected to PLS-DA to identify the differences in VOCs among N-control, W-control, and inoculation fruits (Figure 7). Four groups were clearly discriminated in the treated fruit. The highest-ranking components accounted for 77.1% of the total variance within the data set of VOCs in fruit (Figure 7A). The first component accounted for 50.30% of its total variation, resolved the measured composition profiles of both inoculated fruit from N-control and W-control. *P. expansum* inoculated group was separated from *T. roseum* inoculated one when considering the second component accounted for 26.80% of its total variation. To gain more insights into the VOCs differences among the three groups, the variables important in the projection (VIP) scores were evaluated. Representing a weighted sum of squares of the PLS weight, the VIP with the value >1 is usually considered as important and potential chemical marker to the model being studied. According to Figure 7B, hexanoic acid showed the highest VIP among the treated fruit.

## Discussion

Fruits showed various susceptibilities to different fungi during pathogen–fruit interactions (Louw and Korsten, 2014). In the present study, *P. expansum* produced larger lesions than *T. roseum* (Figure 1). Moreover, inoculations induced ethylene production and respiratory rate, and higher ethylene production and respiratory rate were found in the *P. expansum*-inoculated fruit compared with the *T. roseum* -inoculated ones (Figure 2). These results were similar with Vilanova et al. (2012), who found *P. expansum* had higher infection capacity than *P. digitatum* on “Golden Smoothee” apple. To our knowledge, this is the first report that *P. expansum* was described as a highly aggressive pathogen on apple fruit compared with *T. roseum*. Infected fruit usually showed higher ethylene production and respiratory rate (Yang et al., 2017). Signaling compounds produced during fruit–pathogen interactions could be an important factor contributing to higher ethylene production of fruit. Previous studies have demonstrated that ethylene produced by pathogen upon infection may as a signaling compound in host resistance (Vilanova et al., 2012; Tian et al., 2016). Yang et al. (2017) reported that ethylene produced by *P. digitatum* might activate defense reaction, which prevented further spread of the pathogen and enhance ethylene production during early infection stages in apple fruits. Similar results also found in *P. digitatum*-citrus interaction (González-Candelas et al., 2010). In addition, key enzymes of ethylene biosynthesis of fruit are affected by pathogen during pathogen–fruit interactions (Tatsuki et al., 2009). Some reports indicated that *Penicillium* infection up-regulated expression of *ACS* (1-aminocyclopropane-1-carboxylic acid synthase) that stimulated ethylene production of fruit (Marcos et al., 2005; Vilanova et al., 2012). Wounding caused by pathogen infection and ethylene release of pathogen contribute to ethylene production during fruit–pathogen interactions, but it was not the mainly factor causing higher ethylene release of fruit after infection (Broekaert et al., 2006; González-Candelas et al., 2010; Natalini et al., 2017).

Infection accelerates fruit quality deterioration and softening (Łukaszuk and Ciereszko, 2012; Alkan and Fortes, 2015). In the present study, both of inoculations significantly decreased fruit firmness and the *P. expansum*-inoculated fruit was lower firmness than the *T. roseum* ones (Figure 4A). Higher ethylene release induced by infection promotes expression of cell wall-degrading enzymes in fruit that accelerates firmness decrease by disassembling primary cell wall and middle lamella (Defilippi et al., 2005a). Moreover, extracellular enzymes produced by fungi also lead to a decrease in firmness of fruit (Cantu et al., 2008). TSS and TA contents of fruit were significantly decreased by the two pathogens inoculation (Figure 4B,C). These results are similar with Berger et al. (2004), who noted a decrease in TSS content of tomato after infection. *Alternaria alternata* infection caused lower contents of TSS and TA in muskmelon fruit (Wang et al., 2014). Fructose and malic acid considered as important soluble sugar and organic acid in apple fruit that exhibited a broad range of total sugar and acid levels (Wu et al., 2007). They are also the most important substrates of respiration derived from sugar and acid metabolisms. Infection leading to higher respiratory rate improves the metabolism of the pentose phosphate pathway and tricarboxylic acid cycle, which accelerates carbohydrates and organic acids breakdown (Tang et al., 2010; Tang et al., 2012). Increase of respiration rate stimulated by fungal infection in plant is associated with the improvement of ATP levels, which might be leading to consume a great deal of sugar and acid (Berkowitz et al., 2016; Colombié et al., 2017). Additionally, to satisfy growth demand, fungi consumes carbon sources of host that also decreases the sugar content (Rui and Hahn, 2007). However, compared with *T. roseum*-inoculated fruit, lower TSS content and higher TA content were found in the *P. expansum* ones. Previous studies have reported that pathogens can stimulate secretion of some small molecules such as ammonia and organic acids that modified host pH (Prusky et al., 2013). *P. expansum* modify the host pH by acidification through secreting gluconic acid (Barad et al., 2014), which would contribute to higher TA content and lower TSS content of *P. expansum* fruit. While higher TSS content and lower TA content were found in the *T. roseum* ones, but no report was available about how the pathogen changes pH of host. We hypothesize that alkalization by *T. roseum* modulates the surrounding pH and leads to lower TA and higher TSS content of fruit.

Volatile compounds emitted from fruit are significantly changed by infections. In the present study, content of total VOCs was obviously stimulated by *P. expansum* and *T. roseum* inoculations (Figure 5A). The results are similar with Vikram et al. (2004a,b), who found *P. expansum* infection increased contents of VOCs of “McIntosh,” “Cortland,” and “Empire” apple fruits. Moreover, according to our results, alcohol and aldehyde contents, especially C6 compounds of apple fruit, were notably increased after two pathogens inoculation before 4 DAI (earlier storage); while ester content were increased by inoculations after 6 DAI (middle and later storage) (Figures 5B,C,D, 6 and Table 1). This is in agreement with that found by Encinasbasurto et al. (2017) in tomato inoculated with *A. alternate*. Infection can increase a higher ethylene production of fruit. The association between ethylene and aroma production has verified by using the ethylene action and ethylene biosynthesis inhibitors that result in a reduction in levels of volatiles in apple fruit (Harb et al., 2011). C6 VOCs are derived from lipoxygenase (LOX) pathway, which is involved in lipid oxidation (Noguerol-Pato et al., 2013). This pathway is limited by the level of alcohol acyltransferase (AAT) enzymes, alcohol dehydrogenase (ADH), and LOX enzymes. The three key enzymes activity showed a clear pattern concomitant with ethylene regulation (Defilippi et al., 2005b; Schaffer et al., 2007). Nimitkeatkai et al. (2011) found AAT activity increased in the pathogen-infected apricots fruit and showed a pattern similar to that of ethylene. The activities of LOX and ADH significantly inhibited by 1-MCP treatments, suggesting that ethylene is required for the activity of the corresponding gene or gene product (Harb et al., 2011). Yang et al. (2016) reported that AAT, ADH, and LOX induced significantly and regulated by ethylene and delayed by 1-MCP. Additionally, changes observed in alcohol and aldehyde levels suggest that events upstream of the AAT enzyme step were also under ethylene regulation, including availability of precursors or other enzymatic steps (Yang et al., 2016). Fatty acids of cell membrane are considered to be major precursors of aroma volatiles in apples, are rapidly catabolized by LOX pathway (Song and Bangerth, 2003; Contreras et al., 2016; Wei et al., 2017). With regard to our evaluation, inoculation significantly decreased membrane integrity and increased MDA content (Figure 3). Change in membrane liquidity of fruit is a typical stress response to pathogen attacks (Chun et al., 2009; Yi et al., 2009). It causes loss of membrane integrity in connection with change of fatty acids content. What’s more, higher ethylene release induced by infection increases LOX and phospholipase D, two key enzymes in the proposed membrane oxidation, accelerating damage of cell membrane, which stimulates fatty acids release that actives LOX pathway and leads to emission of LOX-related volatiles (Niinemets et al., 2013; Wei et al., 2017; Shi et al., 2018). Moreover, compared with the *T. roseum*-inoculated fruit, the changes of VOCs, especially C6 VOCs, more affected by *P. expansum* inoculation, and the *P. expansum*-inoculated fruit were significantly distinguished from the *T. roseum*-inoculated ones by volatiles (Figure 6). The results should be related to higher susceptibility of apple fruit to *P. expansum* than *T. roseum*. *P. expansum* inoculation further facilitated ripening, accelerated damage of cell membrane and led to fatty acids release that activated LOX pathway, leading to the emission of LOX-related volatiles.

**Table 1:** Minimum and maximum contents of volatile compounds (μg g^-1^) detected in the two apple cultivars during shelf-life at 20°C.

Volatile compounds	CAS	N-control	W-control	*P. expansum* Inoculation	*T. roseum* Inoculation
***Alcohol***					
2-Methyl-1-butanol	137-32-6	8.46–13.64	9.12–15.17	13.87–28.61	12.06–26.01
1-Hexanol	111-27-3	25.94–31.64	28.43–33.65	20.87–61.47	23.76–58.07
***Aldehyde***					
Hexanal	66-25-1	27.35–36.51	23.59–39.41	32.77–72.24	30.84–67.29
*E*-2-Hexenal	6728-26-3	77.55–121.96	85.86–133.6	83.47–221.09	71.62–218.99
***Ester***					
Ethyl propionate	105-37-3	0.78–3.28	0.73–3.66	1.37–8.89	1.13–8.17
Ethyl butanoate	105-54-4	11.34–18.99	15.88–18.17	20.8–33.45	18.45–29.33
Ethyl 2-methylbutanoate	7452-79-1	14.98–16.87	15.04–19.57	26.56–85.02	20.55–52.11
Butyl acetate	123-86-4	11.24–15.83	15.07–19.15	21.92–39.66	18.82–37.34
2-Methylbutyl acetate	624-41-9	65.44–92.08	78.63–95.32	94.58–189.98	88.97–192.5
Propyl 2-methylbutanoate	37064-20-3	20.73–27.43	20.18–28.78	nd	24.87–58.12
Pentyl acetate	628-63-7	1.96–3.51	2.64–3.72	3.66–6.04	2.13–5.52
Butyl butanoate	109-21-7	15.45–19.76	15.04–22	21.64–42.99	17.81–45.47
Hexyl acetate	142-92-7	118.42–147.87	136.02–164.01	169.05–196.39	149.95–170.37
Propyl hexanoate	626-77-7	21.15–43.47	25.71–46.49	30.16–48.1	27.12–48.54
Pentyl 2-methylbutanoate	68039-26-9	2.05–6.45	1.67–7.39	3.87–11.42	4.97–7.93
Hexyl propanoate	2445-76-3	17.29–55.8	17.04–61.23	21.48–84.8	25.79–88.2
Butyl hexanoate	626-82-4	160.57–193.56	171.52–255.86	201.12–330.17	191.27–276.43
Hexyl 2-methylbutanoate	10032-15-2	486.75–757.08	528.37–683.19	584.19–1012.97	550.83–911.54
2-Methylbutyl hexanoate	2601-13-0	24.41–37.78	24.47–55.72	33.28-77.6	31.89–63.35
*E*-2-Hexenyl butanoate	53398-83-7	2.99–4.17	2.11–3.71	5.96–10.47	4.84–10.81
Propyl octanoate	624-13-5	11.14–24.65	10.87–27.61	18.46-37.11	18.65–32.62
Heptyl 2-methylbutanoate	50862-12-9	3.86–5.68	3.98–6.26	3.15–7.46	3.2–5.14
3-Methylbutyl heptanoate	109-25-1	nd	1.89–3.08	3.06–5.79	2.03–4.52
Hexyl hexanoate	6378-65-0	318.48–397.01	307.54–420.55	489.85–687.97	410.17–567.08
Octanoic acid, 2-methylbutyl ester	nd	26.16–53.11	32.17–65.9	45.26–98.87	42.48–64.55
*E*-2-Hexenyl hexanoate	53398-86-0	3.48–6.95	4.93–11.52	7.9–15.98	7.37–16.95
Butyl heptanoate	5454-28-4	7.14–16.27	10.11–15.28	nd	12.59–18.04
Hexyl octanoate	1117-55-1	34.03–58.11	38.07–65.36	45.2–81.31	37.55–78.42
Butyl decanoate	30673-36-0	5.8–9.3	6.89–8.71	6.4–14.72	nd
Butyl 9-decenoate	nd	7.89–14.84	9.65–17.48	12.3–30.6	12.32–26.34
Ethyl 2,4-decadienoate	3025-30-7	32.63–65.65	37.47–78.77	44.84–100.31	39.82–68.11
Hexyl decanoate	10448-26-7	2.01–4.54	1.92–3.15	2.64–7.71	2.19–4.43
Butyl laurate	106-18-3	2.3–3.87	2.28–5.06	3–6.01	nd
2-Methylbutyl propanoate	2438-20-2	1.48–2.61	1.82–2.98	2.57–3.32	1.88–2.31
Propyl acetate	109-60-4	nd	nd	0–1.28	nd
Hexyl benzoate	6789-88-4	nd	nd	nd	0.46–1.48
***Terpinoide***					
à-Farnesene	502-61-4	84.6–92.74	86.37–93.39	102.2–159.41	91.94–121.87
*E*-β-Farnesene epoxide	83637-40-5	6.45–10.94	10.25–14.93	18.93–27.25	14.13–25.27
*Z,E*-α-Farnesene	26560-14-5	61.46–114.66	66.75–124.53	80.93–186.12	83.32–132.54
***Other***					
2-Methylbutanoic acid	116-53-0	37.7–68.89	54.05–81.23	79.17–99.01	61.65–97.22
Hexanoic acid	142-62-1	3.02–4.6	3.08–4.95	17.01–19.83	15.45–16.64
Undecylenic acid	112-38-9	nd	nd	1.01–3.75	nd
Tridecane	629-50-5	1.62–2.64	1.01–2.98	2–3.94	1.64–3.02
2,6,10-Trimethyl pentadecane	3892-00-0	1.24–3.7	1.99–3.37	2.69–4.4	nd
Heptadecane	629-78-7	3.27–14.14	2.21–18.98	nd	2.51–27.39
Hexadecane	544-76-3	nd	nd	nd	1.51–3.3
Estragole	140-67-0	14.65–51.08	20.01–42.42	24.05–58.18	20.34–53.42
nd: not detected					


Notably, propyl acetate and hexyl benzoate, and undecylenic acid and hexadecane were only identified in the *P. expansum* and *T. roseum* inoculated fruits, respectively. Other studies also have indicated that some VOCs are specific to apple–pathogen interactions (Vikram et al., 2004a,b). However, our results were not similar with others. We hypothesize that it is associated with different apple cultivar and pathogens. Composition of VOCs in different apple cultivars is various, which reveals different response to pathogen attack. Moreover, susceptibilities of fruit to different pathogens affect release of VOCs of fruit. According to PLS-DA, hexanoic acid was the most important factor to distinguish the two pathogens inoculated-fruits from controls (Figure 7B). Some reported demonstrated that hexanoic acid act as a priming defense inducer by activating resistance responses after pathogen attack (Leyva et al., 2008; Vicedo et al., 2009; Kravchuk et al., 2011; Llorens et al., 2013). However, metabolism of these compounds still need to be further studied.

## Conclusion

Both *P. expansue* and *T. roseum* inoculations improved ethylene production and respiratory rate, and accelerated ripening and quality loss of “Red Delicious” fruit. Inoculations increased contents of fruit VOCs, especially C6 VOCs release. Moreover, specific VOCs were only identified in the inoculated fruits. In addition, colonization of “Red Delicious” apples by *P. expansum* showed increased aggressiveness compared to *T. roesum* colonization which followed by promoted differential ripening and VOCs release.

## Data Availability

The datasets generated for this study are available on request to the corresponding author.

## Author Contributions

DG and YB conceived and designed the experiments with the help of DP and YL and wrote the manuscript. DG performed the volatile detection and quantification experiments. DG, YZ, and YH contributed to determination of physiology and quality parameters and achieved statistical and PLS-DA analyses. All authors contributed to the discussion and approved the final manuscript.

## Conflict of Interest Statement

The authors declare that the research was conducted in the absence of any commercial or financial relationships that could be construed as a potential conflict of interest.

## Footnotes

1 www.metaboanalyst.ca
